# Extra-Limites.—Mr. Arnott's Letter to the Editor

**Published:** 1834-07-01

**Authors:** 


### EXTRA-LIMITES.

### To the Editor of the Medico-chirurgical Review.

Sir, The objects of my Essay on the Secondary Effects of Inflammation of the Veins were, an attempt, on the one hand, to shew how death was produced in this disease, and on the other, in what way abscesses originate in distant parts after injuries. The conclusions to which I came were these. I demonstrated that death does not take place from the inflammation of the vein extending to the heart;—I shewed that facts and reasoning tended to establish the conclusion, that the principal cause of the alarming consequences arose from the entrance of pus into the circulation, a similar influence being perhaps also possessed by any inflammatory secretion from the vein; and, finally, I adduced a number of facts, which led me to conclude that the inflammations and abscesses which arise in distant parts after injuries and after parturition, were attributable to the existence of phlebitis in the part of the body primarily affected.

In the last number of your Journal, Dr. Wise pretends that, in some remarks appended to a report of cases of phlebitis treated at St. Bartholomew's Hospital, printed and intended by him for publication in the Medical and Physical Journal for 1827, but withdrawn, proofs will be found of a *resemblance* between his opinions and mine on the subject of phlebitis.

I have referred to the Report alluded to, and which is in your possession. There are no opinions therein expressed resembling mine. The question of the extension or not of the inflammation of the vein to the heart is not considered, much less decided; the entrance of pus or other inflammatory secretion into the circulation is not mentioned; and the occurrence of inflammations and abscesses in distant parts after injuries and after parturition, is neither broached, nor an explanation attempted to be given. Indeed Sir, in that report, no conclusions are drawn—no opinion is expressed as to the primary question of all, the cause of the constitutional affection in phlebitis, and when it is stated that they (the effects) follow "*either* from the structure or the function of the part inflamed," the writer (Dr. Wise) shews that he had formed no opinion, and as regards him, the question is left precisely as it had been by Mr. Hunter, who stated that death took place "*either* from the extension of the inflammation to the heart, or from the entrance of pus into the circulation." And yet this gentleman, Dr. Wise, now talks of having pointed out the cases of phlebitis which occurred at St. Bartholomew's Hospital to the attendants there, as "proofs of the justness of conclusions at which he had previously arrived,"—conclusions which he does not describe, (not even in his two communications to you,) which no one seems to have heard of but himself, and which are not to be found in the report to which he now refers.

Pleuritis as an effect of phlebitis is alluded to in the remarks accompanying the Report, but purulent depositions, affections of the joints, and of the eye, are not mentioned; and as regards pleuritis, no endeavour to account for it is made, so that an opportunity is not afforded of ascertaining, if Dr. Wise had any opinion upon the subject, whether they were not identically the same with those recently promulgated in a country where he had been residing, by M. Velpeau, in his "Memoire sur la Pleurisie a la suite des grandes Opérations Chirurgicales, ou d'un Suppuration plus ou moins abondante."—*Revue Médicale*, 1826.

Unable to support his very general and very vague charge of "appropriation" by any specific or tangible proof, Dr. Wise, in his inability to make out a crime of commission, seeks to help himself by alleging one of omission in the following sentence:—"He, (Mr. Arnott,) was obliged to leave out the peculiar changes in the blood, which *is* so remarkable a consequence, from being aware I had published a note in the Medical and Physical Journal for the year 1827." Now whatever might be my reason for not noticing this change, it certainly was not because Dr. Wise had published "a note"—the change in question had been previously far better described by M. Ribes. "The note" is entitled "Remarks on some of the Phenomena of Inflammation, as they appear on Dissection;" the subject is the proximate cause of inflammation, in which a principal part is given to the capillary veins. When a vein inflames after bleeding, it is "plugged up by consolidated blood some distance above and below the wounded part." "Such a consolidation of blood appears to be a constant effect of a certain degree of inflammation in the branches of veins; and, as the structure and contents of these vessels seem to be the same, may I not be allowed to infer that the same cause will produce the same effect, and that, therefore, a like consolidation of blood takes place in the capillary veins?" Such is Dr. Wise's language. Now let us turn to what was written and published two years previously in a paper entitled, "Exposé succinct des Recherches faites sur la Phlébite, par M. F. Ribes, Révue Médicale, 1825." After stating that in one stage or degree of inflammation the vein may be lined by a false membrane, or that it may be filled with pus, he proceeds "mais quelquefois la veine légèrement enflammée se laisse dilater et distendu par le sang. Ce vaisseau etant malade, ne peut que difficilement, reagir sur le fluide qu'il contient, le sang y stagne, s'y arrete, s'y coagule, se durcit, se dissêche, adhère fortiment et fait, corps avec la veine, alors la membre est plus en moins enflée." P. 11. And two pages farther on, M. Ribes endeavours to shew that the veins are inflamed in erysipelas, which he represents to be essentially seated in the capillary veins. And yet Dr. Wise talks of plagiarism !

From the expression "changes in the blood," although one only is described, but more especially from the tone of his two communications to you, one would almost be disposed, Sir, to infer, that Dr. Wise had other pretensions in view as well as the foregoing, and that he desired it to be supposed that the pathology of the blood equally with inflammation of veins had not previously attracted attention, and was exclusively his own. Besides the paper by M. Velpeau already alluded to, did Dr. Wise never hear of another paper referred to by me in my Essay, and likewise published two years before his note and report? I mean that by M. Bouillaud, "Recherches Cliniques pour servir a l'Historie de la Phlébite, *Revue Médicale*, 1825;" and who, in ascribing the typhoid symptoms in phlebitis to the presence of pus in the system, refers to the experiments of Baglivi, Majendie, and Gaspard, as having produced apparently analogous effects in animals by the injection of acrid and putrid matter into their veins? And does Dr. Wise think that we, living in England, were ignorant of M. Velpeau's "Recherches et observations sur l'altération du sang dans les Maladies, *Revue Médicale*, 1826," and the experiments of M. M. Trousseau, Dupuy, and others?

Dr. Wise states that I suggested to him the publication of a report of the cases of phlebitis, which occurred at St. Bartholomew's Hospital, and that I corrected one if not two of the proof sheets. It would be difficult to correct one sheet even of a report which does not consist of nine pages, but I recollect looking over some part of it at his request, which I presume was made because I had suggested to him as house-surgeon, that an account should be drawn up and published;—(the cases had not then been any where reported.) Neither circumstance however would seem to indicate a disposition on my part to do Dr. Wise a disservice, but directly the reverse; and if this publication of the cases was to give him a pretence to claim opinions which he had not formed, or conclusions which he had not drawn, as well might the Editor of the Lancet, in which they (the cases) subsequently appeared—the late Mr. Rose, of whose paper on depositions of pus and lymph the same cases formed a part—or Mr. Lawrence, in whose practice they occurred, and who communicated them to Mr. Rose, have come forward after the publication of my Essay, and sought to arrogate to themselves the views which I had taken of the cause of the constitutional affection in phlebitis, and of inflammations and abscesses in remote situations after injuries and after parturition.

Dr. Wise states that a very few months after he left England, I presented my Essay to the Medico-Chirurgical Society. The point is of no consequence, but it shows the *animus* and something more. That gentleman left England in June or July, 1827, my Essay was presented to the Society in October, 1828, and had the reading of it been deferred a couple of months, M. Dance would in all probability, and with as much justice as myself, have had to sustain a charge of plagiarism, for in the Archives Générales de Médecine of December, 1828, the first part of that gentleman's paper on uterine phlebitis appeared, in which he expresses opinions almost identical with my own.

In conclusion, Sir, I have to repeat what I formerly asserted, that my views on the subject of phlebitis originated in personal observation of the extraordinary circumstances of the cases I witnessed—not in the suggestions of any one: that the general results and conclusions at which I arrived were formed after a long and laborious investigation of a very extensive subject, and that whatever they may possess of merit or demerit belongs to myself. As regards Dr. Wise, I repeat that I did not derive any materials, facts, or opinions from him—that I never saw a dissection by him of a patient who died from phlebitis, nor do I know what were his opinions thereon, and even now that I have had the advantage of reading his two communications to you I cannot discover what they are.

I have the honor to be, Sir,

Your obedient servant,

*London, May*, 1834. *JAMES M. ARNOTT.*

